# Filaggrin Genotype Determines Functional and Molecular Alterations in Skin of Patients with Atopic Dermatitis and Ichthyosis Vulgaris

**DOI:** 10.1371/journal.pone.0028254

**Published:** 2011-12-02

**Authors:** Mårten C. G. Winge, Torborg Hoppe, Berit Berne, Anders Vahlquist, Magnus Nordenskjöld, Maria Bradley, Hans Törmä

**Affiliations:** 1 Dermatology Unit, Department of Medicine Solna and Center for Molecular Medicine, Karolinska Institutet, Karolinska University Hospital Solna, Stockholm, Sweden; 2 Department of Medical Sciences, Dermatology and Venereology, Uppsala University, Uppsala, Sweden; 3 Department of Molecular Medicine & Surgery and Center for Molecular Medicine, Karolinska Institutet, Karolinska University Hospital Solna, Stockholm, Sweden; University Hospital Hamburg-Eppendorf, Germany

## Abstract

**Background:**

Several common genetic and environmental disease mechanisms are important for the pathophysiology behind atopic dermatitis (AD). Filaggrin (FLG) loss-of-function is of great significance for barrier impairment in AD and ichthyosis vulgaris (IV), which is commonly associated with AD. The molecular background is, however, complex and various clusters of genes are altered, including inflammatory and epidermal-differentiation genes.

**Objective:**

The objective was to study whether the functional and molecular alterations in AD and IV skin depend directly on FLG loss-of-function, and whether *FLG* genotype determines the type of downstream molecular pathway affected.

**Methods and Findings:**

Patients with AD/IV (n = 43) and controls (n = 15) were recruited from two Swedish outpatient clinics and a Swedish AD family material with known *FLG* genotype. They were clinically examined and their medical history recorded using a standardized questionnaire. Blood samples and punch biopsies were taken and trans-epidermal water loss (TEWL) and skin pH was assessed with standard techniques. In addition to *FLG* genotyping, the *STS* gene was analyzed to exclude X-linked recessive ichthyosis (XLI). Microarrays and quantitative real-time PCR were used to compare differences in gene expression depending on *FLG* genotype. Several different signalling pathways were altered depending on *FLG* genotype in patients suffering from AD or AD/IV. Disease severity, TEWL and pH follow FLG deficiency in the skin; and the number of altered genes and pathways are correlated to FLG mRNA expression.

**Conclusions:**

We emphasize further the role of FLG in skin-barrier integrity and the complex compensatory activation of signalling pathways. This involves inflammation, epidermal differentiation, lipid metabolism, cell signalling and adhesion in response to FLG-dependent skin-barrier dysfunction.

## Introduction

Atopic dermatitis (AD; OMIM #605803) is a common chronic, non-contagious, inflammatory skin disorder. Clinical manifestations include early onset of dry skin, pruritus, eczema with typical age-dependent distribution, and personal or family history of atopic disease [1]. Knowledge of the pathophysiology behind the disease is emerging, several common genetic, environmental disease mechanisms and individual trigger factors being of importance [2]. Central in the pathogenesis are combinations of inherited and acquired insults thought to alter epidermal structure. These changes in the physiological skin barrier predispose to increased allergen presentation and are followed by immune activation, which in turn has negative consequences for skin-barrier homeostasis [3]. Impaired homeostasis of the skin leads to increased trans-epidermal water loss (TEWL) and changes in gene expression patterns [4] and enzymatic activity [5].

The most common monogenic disorder of keratinisation, ichthyosis vulgaris (IV; OMIM # 146700), is associated with AD and related atopic manifestations in up to 50% [6]. This contrasts with X-linked recessive ichthyosis (XLI; OMIM # 308100), which is due to mutations in the *STS* gene leading to accumulation of cholesterol sulphate in the stratum corneum. XLI occurs almost exclusively in males and may look almost indistinguishable from IV. However, skin histology and surface pH differ in the two conditions [7] and no association to AD has been reported in XLI. In 2006, it was found that mutations in the *FLG* gene resulting in filaggrin (FLG) dysfunction are the causative genetic factor for IV [8]. Following the frequent co-existence of IV and AD it was also discovered that 20–40% of European and Asian patients with moderate-to-severe AD carry *FLG* mutations. This is so far the most significant genetic finding associated with AD [9]. FLG is important for the structural integrity of the skin, and other functions are attributed to acidic degradation products of FLG, e.g. urocanic acid (UCA) and pyrrolidone carboxylic acid (PCA). These are components of natural moisturizing factors (NMFs) [10] and contributes to maintaining a low pH in the stratum corneum (SC) [11].

In addition to FLG dysfunction, it has previously been demonstrated that the molecular background to the pathogenesis of AD is complex, and that several clusters of genes, including inflammatory and epidermal differentiation [4], [12] are altered in lesional AD skin. We set out to study whether the functional and molecular alterations in AD and IV skin depend directly on FLG loss-of-function variants, and whether the *FLG* genotype determine the type of downstream molecular pathways affected.

## Materials and Methods

### Patient material

Patients (n = 43) with AD (n = 35), AD and IV (n = 5) and IV (n = 3) together with controls (n = 15; subjects without past or present history of AD, dry skin or other atopic manifestations) were identified at the dermatology outpatient clinics at Karolinska University Hospital Solna, Sophiahemmet Stockholm and Uppsala University Hospital; or recruited from a Swedish family material with known *FLG* genotype as described previously [13]. All patients were investigated by a dermatologist performing clinical examination and recording medical history with a standardized questionnaire. Inclusion criteria were: age 18–65 years and diagnosed AD and/or IV. Exclusion criteria were pregnancy; other concomitant skin disease; recent UV-treatment; or recent use of topical or systemic corticosteroids, systemic immunosuppressives or systemic retinoids (<4 weeks). AD was diagnosed according to the UK Working Party's diagnostic criteria and the disease severity for AD was assessed using the scoring atopic dermatitis index (SCORAD) [14]. IV was diagnosed by clinical examination and genetic testing of the *FLG* gene, and in male patients with ichthyosis genetic testing of the steroid sulphatase (*STS*) gene to rule out XLI. Other atopic manifestations such as allergic asthma and allergic rhinoconjunctivitis were assessed through the questionnaire. Blood samples and punch biopsies were taken from all patients and controls. Two 3 mm punch biopsies were obtained from a non-lesional area on each patients forearm, after local anaesthetic with lidocain hydrochloride with adrenalin (Astra Zeneca, Södertälje, Sweden). TEWL was assessed using a Tewameter TM 300 Multi Probe Adapter (Courage+Khazaka electronic GmbH, Köln, Germany) and skin pH was measured using a skin-pH-Meter PH 905 Multi Probe Adapter (Courage+Khazaka electronic GmbH). TEWL and pH were measured from the forearms of patients and controls. The patients were divided into three groups (AD *FLG*+/+; AD *FLG*+/− and AD/IV *FLG*−/−) depending on genotype of the four most prevalent European *FLG*-mutations [6].

#### Subjects for microarray analysis

Five patients from each patient group (AD *FLG*+/+, AD *FLG*+/− and AD/IV *FLG*−/−) were randomly selected for microarray analysis after removing outliers in TEWL and pH. In the AD *FLG*+/− group four were heterozygous carriers of the 2282del4 mutation and one was a heterozygous carrier of the R501X mutation. In the AD/IV *FLG*−/− group four were homozygous carriers of the 2282del4 mutation and one was a homozygous carrier of the R501X mutation. All selected patients had AD. The groups were compared to five healthy controls randomly selected after removing outliers compared to the rest of the control group regarding TEWL or pH. They carried no tested *FLG* mutations.

#### Ethics

The study was conducted according to Declaration of Helsinki principles and was approved by the regional ethics committees at Uppsala University and at Karolinska Institute. All study participants gave written informed consent.

### Genotyping

Genomic DNA was isolated from peripheral blood using QIAamp® DNA mini kit (Qiagen, Hilden, Germany).

#### FLG genotyping

*FLG* genotyping was performed with allelic discrimination in patients and controls for the prevalent European *FLG* mutations R501X, S3247X and R2447X. Genomic DNA was PCR-amplified in 384-well plates. Each well contained 5 ng genomic DNA, 2.5 µl TaqMan Universal PCR Master Mix, 0.125 µl specific Taqman assay solution and 2.375 µl H_2_O. Allelic discrimination was carried out with the ABI PRISM® 7900HT Sequence Detection System and the SDS 2.2.1 sequence detection system program (Applied Biosystems, Stockholm, Sweden). Primers and PCR conditions for tested *FLG* mutations were as described previously [6].

*FLG* mutation 2282del4 was screened for by direct sequencing using an overlapping PCR fragment covering this region [8]. In brief, 50 ng DNA was amplified with 1.25 µl 10 mM dNTPmix (2.5 mM of each), 2.5 µl 10×Rxn buffer - MgCl_2_, 2 µl 50 mM MgCl_2_, 2.5 µl PCR Enhancer, 0.3 µl PlatinumTaq DNA Polymerase (Invitrogen, Lidingö, Sweden), 10.45 µl H_2_0 and 2.5 µl each of forward and reverse primer. Sequencing was analyzed using an ABI® 3730 DNA Analyzer instrument.

#### STS genotyping

Multiplex Ligation-dependent Probe Amplification (MLPA) analysis was run for the *STS* gene using the P160 A2 kit (MRC-Holland, Amsterdam, the Netherlands), as previously described [15] with minor modifications. Typically 100 ng genomic DNA was amplified. The sample was analyzed on the ABI 3130xl Genetic Analyzer. In addition, exon 1–10 of the STS gene was sequenced using primers and PCR conditions previously described in male patients where no deletion was detected [16].

All primer pairs were confirmed specific by database queries (using BLAST and BLAT). The polyphen [17] and the Alamut mutation interpretation software (Interactive Biosoftware, Rouen, France) was used to predict pathogenicity of single nucleotide polymorphisms (SNPs) compared to reference sequence.

### RNA extraction

Skin biopsies were trimmed of subcutaneous fat prior to homogenization. The biopsies were placed in 1 ml Trizol (Invitrogen) and subsequently homogenized using a Polytron homogenizer. Total RNA was isolated as described elsewhere [18]. Total RNA concentration was determined with spectrophotometric analysis and purity was analyzed by the 260∶280 absorbance ratios.

### Microarray analysis

#### Microarray hybridization and scanning

Trizol-extracted total RNA was purified using the RNeasy MiniKit (Qiagen, Valencia, CA). Samples were re-quantified with spectroscopy, and purity was re-analyzed through the 260∶280 absorbance ratios. RNA quality and integrity were assessed and ensured using Bioanalyzer 2100 (Agilent Technologies, Santa Clara, CA) and RNA 6000 NanoAssay. Hybridization was performed with Human Gene 1.0 ST arrays (Affymetrix, Inc, Santa Clara, CA). Briefly, 100 ng of total RNA from each sample was reverse-transcribed to complementary DNA (cDNA) using the Ambion WT Expression kit. The cDNA was subsequently converted to complementary RNA using in vitro transcription with an amplification kit. 10 µg purified complementary RNA was used as a template for another cycle of first-strand cDNA synthesis. Single-stranded cDNA samples were fragmented and end-labeled with the Gene Chip WT cDNA Synthesis Kit (Affymetrix). Approximately 25 ng/µl cDNA was added to the hybridization cocktail, followed by hybridization with the Human Gene 1.0 ST Array GeneChip at 45°C for 16 hours. This was then washed using the Affymetrix Fluidics Station 450. A final step was to measure probe intensities using the GeneChip Scanner 3000. The raw intensity data was normalized using Command Console Software (Affymetrix). The average fluorescence intensity of all annotated genes was calculated using the Robust Multiarray Analysis (RMA) algorithm [19], including a quartile normalization (all arrays are considered to have an equal intensity distribution) and using a background correction for GC-content.

#### Microarray gene expression, data processing, quality control and statistical analysis

The values of individual probes belonging to one probe set were averaged and normalized using Partek Genomics Suite 6.4 (Partek Inc., St. Louis, MO, USA, www.partek.com), from which probes with lowest available p-value and a known GenBank accession ID correspondence were selected for functional analysis. The distribution of the intensity values on the individual arrays was visualized in a signal histogram. One sample was removed due to deviating intensity values compared to the other samples. No other obvious outliers were detected. The intensity values of probe sets specific for the pre-labeled hybridization controls were analyzed and corresponded with the expected values. To check overall data quality, the array contained probe sets for exonic and intronic regions of reference genes (genes thought to be constitutively expressed in many different samples). Their probe set intensities were used to calculate the difference between the area under the curve of the positive and negative probe sets according to the manufacturer's instructions [20].

Genes of interest, all over two-fold up/down-regulated genes (p<0.0005), were analyzed using the Database for Annotation, Visualization and Integrated Discovery functional annotation tool [21] with KEGG pathway analysis.

Functional annotations were also carried out using the Ingenuity Pathway Analysis (IPA; Ingenuity Systems, Redwood City, CA, http://www.ingenuity.com/), in which gene symbols and fold changes of the up- and down-regulated genes were imported.

All microarray data comply with MIAME guidelines and are deposited in ArrayExpress.

#### Identification of enriched cytobands

2292 induced genes and 2076 repressed genes in the AD groups (Table 1) were analyzed for their enrichment in human cytoband regions and gene ontology (GO) terms as defined using the DAVID bioinformatics resources [21], with an individual cutoff for each gene of p<0.0005.

**Table 1:** Number of up- and down-regulated genes in relation to *FLG* genotype in AD and AD/IV patients.

Phenotype and genotype	Upregulated	Downregulated
AD *FLG*+/+	131	181
AD *FLG*+/−	328	429
AD/IV *FLG*−/−	1833	1466
**Total # genes**	**2292**	**2076**

Genes with a minimum two-fold change and p-values<0.0005 were included. Top up- and down-regulated genes for included patients depending on *FLG* genotype. Genes with minimum 2-fold change and p-value<0.0005 were included.

### Quantitative Real-Time PCR

First strand cDNA was synthesized from 1.5 µg total RNA by combining oligo(d)T15, random hexamers, buffer and MMLV-reverse transcriptase (Invitrogen) as previously described [18]. cDNA (5–10 ng total RNA) was subsequently amplified by qPCR using TaqMan® Gene Expression Assays (Applied Biosystems) and TaqMan® Fast Universal PCR Master Mix (2×) in a ABI7500Fast PCR machine (Applied Biosystems). TaqMan gene expression assays used were *FLG* (Hs00856927_g1), ITGA3 (Hs00233722_m1), CD28 (Hs00174796_m1), LAMB3 (Hs00165078_m1), CTNNA1 (Hs00944792_mH), WAS (Hs00166001_m1), JAM2 (Hs01022013_m1), ITGAE (Hs00559580_m1), PTK2B (Hs00169444_m1), TLR2 (Hs00152932_m1), STAT2 (Hs01013123_m1), with ubiquitination factor E4A (Hs01083625_m1), 18S ribosomal RNA 1 (Hs03928985_g1) and GAPDH (Hs02758991_g1) used as endogenous controls. Expression levels were measured in duplicate. For genes with expression below the C_T_ fluorescence threshold, C_T_ was set at 40 to calculate the relative expression. Analysis was performed using an ABI PRISM 7500Fast sequence detection system (Applied Biosystems).

### Statistical analysis

To identify differentially expressed genes between the different experimental groups in the microarray analysis, a two-way analysis of variance (*ANOVA*) was performed for each patient group compared to the healthy control group using Partek Genomics Suite 6.4. For each comparison between two experimental groups the fold change of every annotated gene, together with their corresponding p-value, was exported to Microsoft Office Excel. For quantitative Real-Time PCR, the relative mRNA expression and statistical significance were calculated using the REST 2009 software (available at www.qiagen.com) using *Fisher's exact test*. For genes chosen for pathway analysis, significance was corrected with *Bonferroni multiple testing*. Statistical significance for SCORAD was calculated using *student's t -test*. P-values<0.05 were considered as significant.

## Results

### Genotyping and clinical presentation

Among the included patients (n = 43) fourteen carried none of the prevalent *FLG* mutations tested and were included in the AD *FLG*+/+ group. Fourteen AD patients carried one prevalent heterozygous FLG mutation (one R501X; thirteen 2282del4) and were included in the AD *FLG*+/− group. Fifteen AD/IV patients carried either a homozygous or a compound heterozygous *FLG* mutation (two R501X; nine 2282del4; one S3247X; two 2282del4/S3247X and one 2282del4/R501X) and were included in the AD/IV *FLG*−/− group (of these fifteen patients, three had IV phenotype without AD at the time of examination). One patient was excluded from the AD/IV FLG−/−group after no *FLG* mutations were detected, and subsequent *STS* genotyping revealed a point mutation, recently published elsewhere [16]. In the AD *FLG*+/+ group 78.6% were females, the average age was 56 (range 28–78) and the mean SCORAD was 7.6 (range 0–14.7). In the AD *FLG*+/− group, 64.3% were female, the average age was 54 (range 28–71) and the mean SCORAD was 15.4 (range 6.2–25.8). For the AD/IV *FLG*−/− group 46.7% were female, the average age was 59 (range 44–70) and the mean SCORAD for AD patients in this group was 14.1 (range 7–44.5). The AD *FLG*+/+ had significantly lower SCORAD than the AD *FLG*+/− and the AD patients in the AD/IV *FLG*−/− group (p = 0.02). The control group consisted 43% females and the average age was 52 (range 24–75).

### TEWL and pH

**Figure 1:**
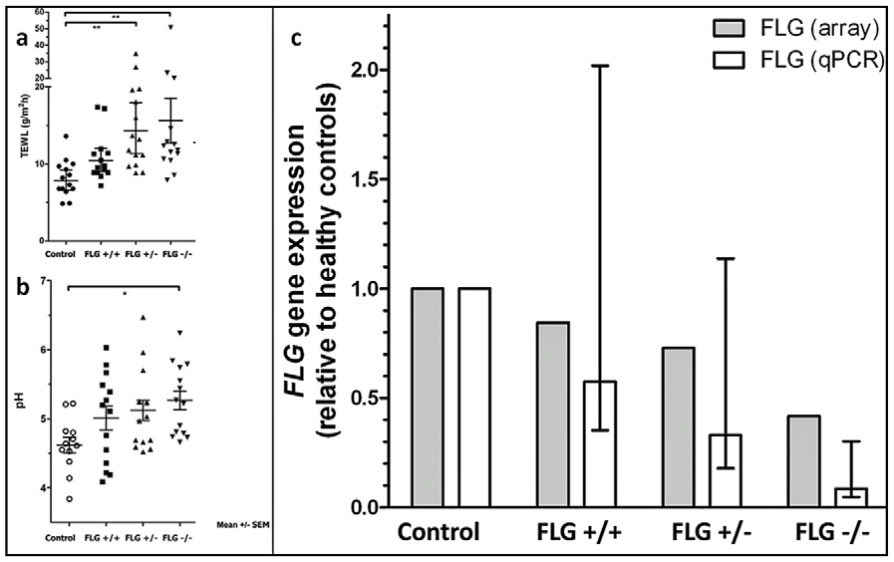
Mean trans-epidermal water loss (TEWL) (a) and pH (b) and decrease in mRNA expression (c) in the AD *FLG*+/+, AD *FLG*+/− and the AD/IV *FLG*−/− group. All groups compared to a healthy control group. Significant changes are denoted with * and ** (p<0.05 and p<0.01), respectively. All groups had significantly altered FLG expression compared to the healthy control group with qPCR; for the *FLG*+/+ p = 0.04, the *FLG*+/− p = 0.001 and the *FLG*−/− group p = 0.001. From the array expression results the *FLG*+/+ group was lower but not significant (p = 0.59) whereas the expression was significantly lower in the *FLG*+/− (p = 0.04) and *FLG*−/− groups (p = 0.000008).

Significantly higher TEWL was observed in the AD/IV *FLG*−/− and AD *FLG*+/− than in the healthy control group. The mean TEWL was higher also in the AD *FLG*+/+ group, although this did not reach statistical significance. pH was significantly higher in the AD/IV *FLG*−/− group than in the healthy control group. Mean pH was higher also for the AD *FLG*+/− and *FLG*+/+ groups, although this did not reach statistical significance (Fig. 1a).

### FLG mRNA expression depending on genotype

All patient -groups showed lower mRNA expression of *FLG* than the control group, both with microarray analysis and with quantitative real-time PCR (qPCR). The mRNA expression levels were lowest in the *FLG*−/− group (array p = 0.000008; qPCR p = 0.001), but significantly reduced also in the *FLG*+/− (array p = 0.04; qPCR p = 0.001) and with qPCR also in the *FLG*+/+ group (array p = 0.59; qPCR p = 0.04) than in the healthy control group (Fig. 1b).

### Altered expression profiles in AD

The microarrays representing 28869 annotated genes with 764885 distinct probes were used to identify and compare the gene expression of AD skin compared to healthy skin, and the difference in expression pattern depending on *FLG* genotype. The design of the Human Gene 1.0 ST array was based on the March 2006 (UCSC hg18, NCBI Build 36) human sequence assembly, containing over 99 percent coverage of sequences present in the RefSeq database. A full list of significantly altered genes is provided as Table S1. Among these, the Partek Genomics suite 6.4 was used to detect 4368 differentially expressed genes (minimum 2-fold change and p-value<0.0005) (Table 1). These differentially expressed genes were distributed according to the chromosomal enrichment illustrated in Table S2.

### Distribution of differentially expressed genes

**Figure 2:**
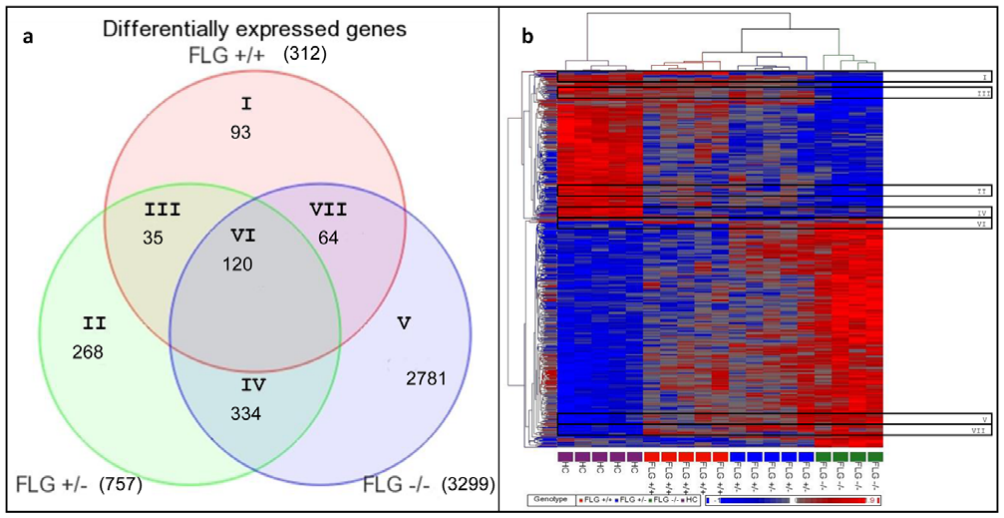
Top overlapping differentially expressed genes in AD skin (a) and heat map of transcriptional levels of genes in AD skin and controls (b). Genes with altered expression in patients with *FLG* wild type genotype *(FLG+/+)*, heterozygote *FLG* mutation genotype *(FLG+/−)*, and homozygote *FLG* mutation genotype *(FLG−/−)*. Clusters containing differentially expressed genes in marked regions I–VII are corresponding regions in a) and b). A list of p-values and fold changes for all differentially expressed genes are described in Table S1. Hierarchal clustering analysis was performed in both the gene (row) and experiment (column) dimension. Contrast value for each gene is shown, e.g. the standardized mean difference between the gene's expression in the group versus overall expression.

The distribution of differentially expressed genes in all patient groups depends on their *FLG* genotype (Fig. 2a). Hierarchical clustering was used to group these differentially expressed genes, based on similarity in expression across the samples and to group individuals on the basis of similarities in gene-expression patterns (Fig. 2b). Each column represents a single array experiment and clusters from Fig. 2a are marked I–VII, respectively.

#### Altered pathways for cellular development and differentiation, inflammatory response and cell-to-cell signaling in AD/IV skin compared to healthy controls, regardless of FLG status

**Figure 3:**
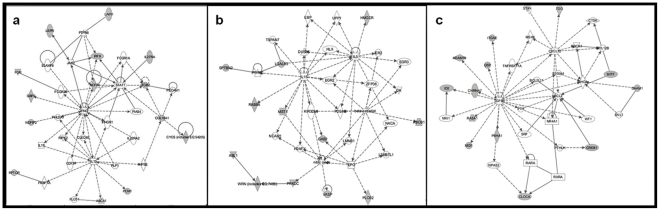
Ingenuity Pathways Analysis showing differentially expressed genes mapped to pathway for inflammatory response (a) and cellular development and differentiation (b and c) for all patient groups. Each gene mapped to this pathway (marked in grey) show significant altered expression to this pathway (p-value<0.0005).

Ingenuity Pathway Analysis of differentially expressed genes for all patient groups reveals a pathway mapped to inflammatory response that was significantly induced compared to the control group (Fig. 3). In addition, there were several altered pathways mapped to cellular development and differentiation compared to healthy controls (Fig. 3).

#### Significantly altered pathways depending on FLG genotype status

In AD/IV skin with *FLG*−/− genotype, several pathways were significantly altered compared to the healthy control group. Focal adhesion, extracellular matrix receptor interaction, regulation of actin cytoskeleton and calcium signaling pathways showed significantly altered expression (Table 2).

**Table 2:** Top altered molecular pathways depending on *FLG* genotype.

AD *FLG*+/+	# genes	p-value
hsa04510:Focal adhesion	30	0.001
hsa04810:Regulation of actin cytoskeleton	29	0.006
hsa04512:ECM-receptor interaction	14	0.02

AD *FLG*+/−	# genes	p-value
hsa04512:ECM-receptor interaction	29	0.000005**
hsa04510:Focal adhesion	52	0.000009**
hsa02010:ABC transporters	19	0.00001**
hsa04810:Regulation of actin cytoskeleton	47	0.002*
hsa04020:Calcium signaling pathway	37	0.01
hsa04660:T cell receptor signaling pathway	23	0.04
hsa04520:Adherens junction	17	0.07

AD/IV *FLG*−/−	# genes	p-value
hsa04070:Phosphatidylinositol signaling system	54	0.0000008**
hsa04510:Focal adhesion	118	0.0000008**
hsa04512:ECM-receptor interaction	57	0.000002**
hsa04810:Regulation of actin cytoskeleton	123	0.000003**
hsa04020:Calcium signaling pathway	100	0.00004*
hsa04660:T cell receptor signaling pathway	64	0.0003
hsa02010:ABC transporters	31	0.0003
hsa04530:Tight junction	71	0.007

All included molecules were selected from Table S1 with matching inclusion criteria and mapped using KEGG Pathway analysis. Bonferroni corrected p-values<0.05 are indicated by * and Bonferroni corrected p-values<0.005 are indicated by **. Candidate genes mapped to each pathway are outlined in Table S3.

In AD skin with *FLG*+/− genotype, focal adhesion and extracellular matrix receptor interaction pathways displayed, similarly to the *FLG*−/− group, a significant deviation compared to the healthy controls, together with ABC transporting pathway and actin cytoskeleton regulation (Table 2).

For AD skin without *FLG* mutations focal adhesion, ECM receptor interaction and regulation of actin cytoskeleton show a deviating trend (Table 2), but this was not statistically significant. For a list of candidate genes mapped to altered pathways depending on *FLG* genotype see Table S3.

### Quantitative Real-Time PCR

#### Identification of candidate genes

For complimentary testing of selected significantly differentially expressed genes mapped in the pathway analysis qPCR was performed from genes in cytobands previously associated to AD (Table S2 and Table S3) and with fold changes close to two-fold up- or down-regulation. Of significantly altered pathways, *ITGA3* and *CTNNA1* were mapped to focal adhesion, *LAMB3* to extracellular matrix receptor interaction, *ITGAE* to actin cytoskeleton, *JAM2* to tight junction pathway, *VAV1* and *CD28* to T cell receptor signaling pathway, and, *PTK2B4* was mapped to calcium signaling pathway; all mapped using DAVID bioinformatics resources [21] with the KEGG pathway analysis option. *TLR2* and *STAT2* represent genes associated with immunological response mapped using Ingenuity Pathways Analysis (Fig. 3).

#### Results of Quantitative Real-Time PCR

To study the relative expression of candidate genes, qPCR was performed in 40 patients and 13 controls. FLG was significantly down-regulated in all patient groups (*FLG*+/+ p = 0.043; *FLG*+/− p = 0.001; *FLG*−/− p = 0.001) (Fig. 2). *CD28* (p = 0.007), *CTNNA1* (p = 0.003) and *LAMB3* (p = 0.01) were all significantly altered in the AD *FLG*+/+ group. *STAT2* (p = 0.001) was significantly altered in the AD *FLG*+/− whereas *STAT2* (p = 0.01), *CTNNA1* (p = 0.001), *JAM2* (p = 0.001) and *CD28* (p = 0.001) all were significantly altered in the AD/IV *FLG*−/− group. Further details regarding qPCR expression for these genes are given in Table S4.

## Discussion

FLG was shown to be down-regulated both by microarray analysis and qPCR in all AD/IV groups compared to the healthy controls. Although there was a gradient of down-regulation depending on *FLG* genotype with the lowest FLG expression in patients with *FLG*−/− genotype followed by the *FLG*+/− genotype, the *FLG*+/+ group also displayed down-regulation compared to healthy control skin. Recent studies have shown that pro-inflammatory cytokines may modulate the expression of FLG, even in patients without *FLG* mutations, which might be one of the underlying explanations of our finding [22], [23].

Many of the potential AD candidate genes significantly altered in our study were located in chromosomal regions previously linked to AD [24] (Table S2), further highlighting these regions as interesting loci for potential candidate genes involved in AD susceptibility. The distributions of these differentially expressed genes in our study depended on *FLG* genotype, where several clusters were unique for each group, and others overlapped (Fig. 2). Genes from these groups are mapped to significantly altered pathways in each patient group. The functional alterations evident from the significantly higher TEWL and pH (Fig. 1) in the FLG-deficient groups may influence the number of induced or repressed genes involved in tightly regulated processes such as inflammatory response following a more permeable barrier, as well as enzymatic activity where the pH level is important [11].

The importance of changes in TEWL and pH has recently been highlighted in FLG deficient skin; where reduced levels of FLG degradation products are proposed to increase TEWL and pH; decreasing stratum corneum hydration and altering enzymatic activity [25], [26]. This may account for alterations in corneocyte and lipid organization within the SC [26]. Given the frequent phenotypic overlap between dry skin, IV and AD (evident in our *FLG*−/− group as well); it is proposed that these functional alterations are important for the pathogenesis in both IV and AD skin with FLG deficiency. In support of this hypothesis, our AD patients without *FLG* mutations displayed lower functional barrier impairment measured by TEWL, lower pH and significantly lower mean SCORAD than AD patients with *FLG* mutations (*FLG*-repeat variation may also influence the phenotype [27]. However, we did not investigate this). In addition, the lowest number of significantly altered genes was detected in our AD *FLG*+/+ group. This suggests a correlation between number of affected genes, barrier impairment and disease severity among included AD patients.

Of the many genes previously associated to AD [24] several were also dysregulated in our array data, such as serine protease inhibitor kazal-type 5 (*SPINK-5*), mast cell chymase (*CMA1* and interleukin 4 *(IL-4)* (Table S1). Any discrepancies regarding expression of inflammatory mediators commonly found in AD may at least in part be due to lower expression of these genes in non-lesional skin. *CD28* and *STAT2* are two inflammatory markers that were confirmed to be altered also by qPCR. *CD28* has been suggested to be involved in the inflammatory response in AD [28] and *STAT2* has been described as a candidate gene involved in mediating pro-inflammatory cytokines [29]. In addition, genes mapped to adhesion such as *CTNNA1*, *JAM2* and *LAMB3* were also confirmed to be significantly altered. Defects in cell adhesion have recently been highlighted as important in AD pathogenesis, with the finding of impairment in tight junctions contributing to the barrier dysfunction and immune dysregulation [30]. Down-regulation of tight-junction proteins such as occludin and ZO-1 has also been demonstrated in IV skin recently [26] and tallies with our gene-expression pattern in FLG-deficient skin (Table S1). The *LAMB3* gene encodes laminin-5, a glycoprotein that anchors basal cells to the underlying basal membrane [31], whereas *CTNNA1* and *JAM2*, in addition to their cell adhesion function, have been suggested to be involved in cell differentiation [32] and lymphocyte homing [33], respectively. Interestingly *CTNNA1* and *LAMB3* were significantly altered in the AD group without *FLG* mutations. The underlying explanation could in part be the effects of putative down-regulation of FLG also in this group, but as the expression of *LAMB3* did not reach significance in the AD *FLG*−/− group and neither did *CTNNA1* or *LAMB3* in the AD *FLG*+/− group, other explanations are plausible, including that these genes are candidates for the primary pathogenesis in AD in addition to FLG deficiency.

The molecular mechanisms involved in the phenotype of AD following the functional barrier impairment in our material involve altered pathways such as cytoskeleton structure, calcium- and phospatidylinositol signaling and ATP binding cassette (ABC) transport system (Table 2). It has been suggested that FLG is of importance for cytoskeleton organization by aggregating keratin intermediate filaments (KIFs); and that FLG deficiency may cause cytoskeleton abnormalities such as perinuclear keratin retraction in granular cells [26]. KIF polymerization is actin-dependent [34] and subsequently actin-cytoskeleton aberrations may contribute to the peripheral KIF retraction previously demonstrated in FLG-deficient skin [26]. However, the role of FLG in impaired intermediate filament aggregation has been challenged [35] and other factors than FLG deficiency may explain the alterations in the pathway for the regulation of the actin cytoskeleton. Our findings support this, as pathways for actin-cytoskeleton regulation were altered in all our patient groups including the group without *FLG* mutations. In addition, several keratins (including *KRT1* and *KRT10*) were significantly down-regulated in AD patients both with and without *FLG* mutations (Table S1). As the actin filament system has been suggested to be involved in KIF transport [34], it is possible that increased actin cytoskeleton regulation is a compensatory mechanism following a lower keratin expression. Altogether, our data suggests that both keratin expression and KIF regulation are subject to modulation in AD skin independently of *FLG* mutations.

FLG may be involved in calcium metabolism in the skin [36], and the calcium gradient is important for epidermal differentiation - a loss of this gradient increases keratinocyte proliferation and decreases differentiation [37]. Impaired calcium metabolism has been demonstrated in other conditions where the skin barrier is disrupted, such as Hailey-Hailey disease [38] and in psoriatic skin [39]. Further, defective lipid transportation and defects in lamellar body extrusion have previously been reported in AD [40], [41] and mutations in this pathway may cause severe ichthyotic conditions such as Harlequin Ichthyosis [42]. Our FLG-deficient groups show alterations both in the pathway for calcium signaling and for ABC transport system, indicating that alterations in these pathways are involved in the pathogenesis of IV and AD with FLG deficiency.

In conclusion, we have demonstrated that several functional and molecular mechanisms *in vivo* in patients suffering from AD and IV depend on *FLG* genotype. Disease severity of AD, the gradient of TEWL and pH follow loss of FLG expression in the skin; and the number of altered genes and pathways may be correlated to FLG mRNA expression. We here emphasize further the role of FLG for the functional integrity of the skin barrier and the complex subsequent signaling systems involving inflammation, epidermal differentiation, lipid metabolism, cell signalling and adhesion that are affected in response to FLG deficiency.

## Supporting Information

Table S1 **Human Gene 1.0 ST array mRNA expression.** P-values and fold change of each annotated gene are mean values of five subjects from each patient group (*FLG*+/+, *FLG*+/− and *FLG*−/−) compared to a five subjects from the healthy control group (using the March 2006: UCSC hg18, NCBI Build 36).
(XLSX)

Table S2 **Enrichment of chromosomal regions in all AD patients.** Chromosomal regions (cytobands) enriched in 2292 induced genes and 2076 repressed genes using DAVID bioinformatics resource. Cytobands are sorted by p-value and previously described genetic association to AD is marked yellow.
(DOCX)

Table S3 **Candidate genes mapped to altered pathways depending on** ***FLG*** **genotype.** Each annotated gene with corresponding p-value and fold change depending on *FLG* genotype and corresponding cytoband. Cytobands with previously reported AD association marked with yellow.
(DOCX)

Table S4 **Quantitative Real-Time PCR mRNA expression depending on** ***FLG*** **genotype.** Selected genes with corresponding p-values and ratio of up- or down regulation in patient groups depending on *FLG* genotype compared to a healthy control group.
(XLS)
