# Supplementary material for: Filaggrin Genotype Determines Functional and Molecular Alterations in Skin of Patients with Atopic Dermatitis and Ichthyosis Vulgaris
Source: PLoS One. 2011 Dec 2;6(12):e28254. doi: 10.1371/journal.pone.0028254 (PMC3229525; doi:10.1371/journal.pone.0028254)
Supplement: Table S2 — Enrichment of chromosomal regions in all AD patients. Chromosomal regions (cytobands) enriched in 2292 induced genes and 2076 repressed genes using DAVID bioinformatics resource. Cytobands are sorted by p-value and previously described genetic association to AD is marked yellow. (DOCX) [file pone.0028254.s002.docx]

| **Enriched** | **# of cytoband** | **p-value** | **Fold** |  | **Enriched** | **# of cytoband** | **p-value** | **Fold** |
| --- | --- | --- | --- | --- | --- | --- | --- | --- |
| **cytoband** | **genes altered** |  | **enrichment** |  | **cytoband** | **genes altered** |  | **enrichment** |
| 19p13.3 | 156 | 4,79E-18 | 1,77 |  | 1p13.3 | 40 | 2,59E-05 | 1,77 |
| 16p13.3 | 149 | 1,11E-14 | 1,69 |  | 11q23.3 | 45 | 3,10E-05 | 1,70 |
| 19q13.4 | 57 | 1,60E-12 | 2,18 |  | 7q22.1 | 60 | 3,12E-05 | 1,57 |
| 16q22.1 | 74 | 1,72E-11 | 1,92 |  | 3q13.2 | 21 | 4,63E-05 | 2,20 |
| 19p13.11 | 55 | 4,52E-11 | 2,10 |  | 9q34 | 35 | 5,55E-05 | 1,80 |
| 11q13 | 76 | 1,19E-10 | 1,85 |  | 3p21 | 23 | 5,69E-05 | 2,10 |
| 19q13.2 | 92 | 1,37E-08 | 1,64 |  | 2p22-p21 | 14 | 6,30E-05 | 2,64 |
| 12q13 | 50 | 1,59E-08 | 1,97 |  | 3p21.1 | 34 | 9,85E-05 | 1,78 |
| 19p13.2 | 108 | 2,61E-08 | 1,56 |  | 16q13 | 31 | 1,13E-04 | 1,83 |
| 3p21.3 | 40 | 3,62E-08 | 2,10 |  | 1p36.11 | 39 | 1,17E-04 | 1,70 |
| 5q31 | 68 | 2,74E-07 | 1,69 |  | 4p12 | 16 | 1,29E-04 | 2,38 |
| 11p15.5 | 73 | 3,02E-07 | 1,65 |  | 1q22-q23 | 16 | 1,29E-04 | 2,38 |
| 19q13.1 | 39 | 3,54E-07 | 2,01 |  | 17q12 | 51 | 1,44E-04 | 1,57 |
| 12q24.31 | 51 | 3,81E-07 | 1,83 |  | 8q24.3 | 64 | 1,58E-04 | 1,49 |
| 17q25 | 32 | 4,17E-07 | 2,16 |  | 15q24 | 17 | 1,59E-04 | 2,29 |
| 2q35 | 47 | 7,44E-07 | 1,85 |  | 6p21.1 | 42 | 2,17E-04 | 1,63 |
| 19q13.42 | 41 | 7,60E-07 | 1,93 |  | 17q21 | 36 | 2,28E-04 | 1,70 |
| 17p13.1 | 65 | 8,26E-07 | 1,67 |  | 7q22 | 23 | 2,42E-04 | 1,97 |
| 1q21 | 55 | 9,51E-07 | 1,75 |  | 21q22.3 | 72 | 2,97E-04 | 1,43 |
| 1q32 | 34 | 1,17E-06 | 2,05 |  | 6p21.3 | 134 | 3,26E-04 | 1,29 |
| 2p23.3 | 42 | 1,45E-06 | 1,89 |  | 19p13.1 | 30 | 3,40E-04 | 1,77 |
| 11q13.1 | 59 | 1,95E-06 | 1,69 |  | 17q25.3 | 59 | 3,45E-04 | 1,48 |
| 17q11.2 | 65 | 1,96E-06 | 1,64 |  | 11q23 | 26 | 4,17E-04 | 1,84 |
| 12p13 | 41 | 2,70E-06 | 1,87 |  | Xp11.23 | 43 | 4,28E-04 | 1,58 |
| 19q13.3 | 57 | 3,50E-06 | 1,68 |  | 1q25 | 19 | 4,47E-04 | 2,07 |
| 12q24.11 | 25 | 5,46E-06 | 2,21 |  | 12p13.3 | 21 | 4,65E-04 | 1,98 |
| 19p13.3-p13.2 | 18 | 6,97E-06 | 2,55 |  | 4p16.3 | 42 | 4,69E-04 | 1,59 |
| 9q34.3 | 64 | 1,05E-05 | 1,59 |  | 2p23 | 13 | 5,26E-04 | 2,45 |
| 14q24.3 | 54 | 1,05E-05 | 1,66 |  | 17q12-q21 | 29 | 5,91E-04 | 1,75 |
| 10q24 | 25 | 1,33E-05 | 2,14 |  | 12q24.1 | 14 | 6,49E-04 | 2,33 |
| 17q25.1 | 43 | 1,33E-05 | 1,76 |  | 8p21 | 15 | 7,46E-04 | 2,24 |
| 17p13.2 | 40 | 1,53E-05 | 1,80 |  | 20q13.3 | 15 | 7,46E-04 | 2,24 |
| 22q13.1 | 50 | 2,17E-05 | 1,67 |  | 5q31.1 | 31 | 8,09E-04 | 1,69 |
| 9q34.1 | 18 | 2,44E-05 | 2,43 |  | 11p15.3 | 16 | 8,17E-04 | 2,16 |
| 1p34 | 23 | 2,48E-05 | 2,17 |  | 17q21.2 | 30 | 8,75E-04 | 1,70 |
