# Supplementary material for: Filaggrin Genotype Determines Functional and Molecular Alterations in Skin of Patients with Atopic Dermatitis and Ichthyosis Vulgaris
Source: PLoS One. 2011 Dec 2;6(12):e28254. doi: 10.1371/journal.pone.0028254 (PMC3229525; doi:10.1371/journal.pone.0028254)
Supplement: Table S3 — Candidate genes mapped to altered pathways depending on FLG genotype. Each annotated gene with corresponding p-value and fold change depending on FLG genotype and corresponding cytoband. Cytobands with previously reported AD association marked with yellow. (DOCX) [file pone.0028254.s003.docx]

| **Focal adhesion** |  | *FLG* +/+ |  | *FLG* +/- |  | *FLG* -/- |  |  |
| --- | --- | --- | --- | --- | --- | --- | --- | --- |
| Gene Symbol | RefSeq | p-value | fold change | p-value | fold change | p-value | fold change | Cytoband |
| ITGA2 | NM_002203 | 5.37E-07 | -6.52 | 6.09E-08 | -8.77 | 6.43E-08 | -9.92 | 5q11.2 |
| ITGA3 | NM_002204 | 0.02 | 1.89 | 0.0005 | 2.99 | 0.0004 | 3.24 | 17q21.33 |
| CTNNB1 | NM_001904 | 0.01 | -2.22 | 0.02 | -2.08 | 3.98E-06 | -6.63 | 3p21 |
|  |  |  |  |  |  |  |  |  |
| **T cell receptor** |  | *FLG* +/+ |  | *FLG* +/- |  | *FLG* -/- |  |  |
| Gene Symbol | RefSeq | p-value | fold change | p-value | fold change | p-value | fold change | Cytoband |
| VAV1 | NM_005428 | 0.28 | 1.26 | 0.14 | 1.38 | 0.00001 | 3.76 | 19p13.2 |
| VAV2 | NM_001134398 | 0.25 | 1.29 | 0.0002 | 2.67 | 0.0002 | 2.80 | 9q34.1 |
| LCP2 | NM_005565 | 0.004 | 1.84 | 0.02 | 1.57 | 0.00003 | 2.89 | 5q33.1-qter |
| CD38 | CR610722 | 0.01 | 1.23 | 0.0006 | 1.36 | 0.00003 | 1.54 | 4p15.32 |
| IL2 | NM_000586 | 0.001 | 1.52 | 0.0005 | 1.59 | 0.001 | 1.56 | 4q26-q27 |
| CD28 | NM_006139 | 0.46 | 1.12 | 0.007 | 1.59 | 0.0041 | 1.70 | 2q33.2 |
|  |  |  |  |  |  |  |  |  |
| **ECM Receptor interaction** | | *FLG* +/+ |  | *FLG* +/- |  | *FLG* -/- |  |  |
| Gene Symbol | RefSeq | p-value | fold change | p-value | fold change | p-value | fold change | Cytoband |
| COL5A3 | NM_015719 | 0.19 | -1.38 | 0.005 | -2.10 | 0.01 | -2.02 | 19p13.2 |
| COL11A2 | NM_080680 | 0.22 | 1.40 | 0.16 | 1.47 | 0.03 | 1.90 | 6p21.3 |
| COL4A4 | NM_000092 | 0.047 | 1.28 | 0.06 | 1.27 | 0.004 | 1.48 | 2q35-q37 |
| LAMB3 | NM_000228 | 0.095 | 2.00 | 0.03 | 2.49 | 0.04 | 2.54 | 1q32 |
|  |  |  |  |  |  |  |  |  |
| **Adherens junction** |  | *FLG* +/+ |  | *FLG* +/- |  | *FLG* -/- |  |  |
| Gene Symbol | RefSeq | p-value | fold change | p-value | fold change | p-value | fold change | Cytoband |
| ACTN4 | NM_004924 | 0.008 | -2.95 | 0.34 | -1.43 | 0.0002 | -5.95 | 19q13 |
| CTNNA1 | NM_001903 | 0.007 | -4.57 | 0.0009 | -7.11 | 0.002 | -6.43 | 5q31.2 |
| CTNNB1 | NM_001904 | 0.11 | -1.97 | 0.45 | -1.36 | 0.0001 | -8.07 | 3p21 |
| WAS | NM_000377 | 0.01 | 1.32 | 0.16 | 1.26 | 0.0001 | 2.32 | Xp11.4-p11.21 |
| WASL | NM_003941 | 0.006 | -2.04 | 0.005 | -2.07 | 0.01 | -1.97 | 7q31.3 |
|  |  |  |  |  |  |  |  |  |
| **Tight junction** |  | *FLG* +/+ |  | *FLG* +/- |  | *FLG* -/- |  |  |
| Gene Symbol | RefSeq | p-value | fold change | p-value | fold change | p-value | fold change | Cytoband |
| MAP3K6 | NM_004672 | 0.39 | 1.12 | 0.19 | 1.19 | 0.003 | 1.59 | 1p36.11 |
| CADM3 | NM_021189 | 0.006 | 2.14 | 0.087 | 1.55 | 0.10 | 1.57 | 1q21.2-q22 |
| ICAM3 | NM_002162 | 0.02 | 1.81 | 0.0006 | 2.71 | 0.0001 | 3.63 | 19p13.3-p13.2 |
| JAM2 | NM_021219 | 0.32 | -1.35 | 0.051 | -1.83 | 0.0002 | -4.00 | 21q21.3 |
| CLDN1 | NM_021101 | 0.32 | -1.36 | 0.18 | -1.53 | 0.002 | -3.13 | 3q28-q29 |
| CLDN5 | NM_001130861 | 0.10 | 1.22 | 0.002 | 1.53 | 0.008 | 1.44 | 22q11.21 |
| CLDN19 | NM_148960 | 0.24 | -1.21 | 0.001 | -1.79 | 0.002 | -1.78 | 1p34.2 |
| OCLN | NM_002538 | 0.02 | -1.72 | 0.005 | -1.90 | 0.0001 | -2.77 | 5q13.1 |
|  |  |  |  |  |  |  |  |  |
| **Regulation of actin cytoskeleton** | | *FLG* +/+ |  | *FLG* +/- |  | *FLG* -/- |  |  |
| Gene Symbol | RefSeq | p-value | fold change | p-value | fold change | p-value | fold change | Cytoband |
| NCKAP1L | NM_005337 | 0.18 | -1.39 | 0.004 | -2.16 | 0.0003 | -3.04 | 12q13.13 |
| INSRR | NM_014215 | 0.18 | -1.55 | 0.003 | -2.91 | 0.0004 | -4.13 | 1q21-q23 |
| DIAPH1 | NM_005219 | 0.004 | -5.24 | 0.004 | -5.05 | 0.0005 | -9.02 | 5q31 |
| ITGAD | NM_005353 | 0.04 | -1.54 | 0.0006 | -2.27 | 0.0007 | -2.35 | 16p13.1-p11 |
| ITGAE | NM_002208 | 0.26 | 1.33 | 0.08 | 1.58 | 0.003 | 2.45 | 17p13 |
| ITGAX | NM_000887 | 0.70 | 1.07 | 0.03 | 1.55 | 0.015 | 1.68 | 16p11.2 |
|  |  |  |  |  |  |  |  |  |
| **Calcium Signaling pathway** | | *FLG* +/+ |  | *FLG* +/- |  | *FLG* -/- |  |  |
| Gene Symbol | RefSeq | p-value | fold change | p-value | fold change | p-value | fold change | Cytoband |
| ATP2B4 | NM_001001396 | 0.30 | -1.22 | 0.05 | -1.48 | 0.0006 | -2.29 | 1q32.1 |
| PTK2B | NM_173174 | 0.70 | 1.13 | 0.07 | 1.83 | 0.005 | 2.89 | 8p21.1 |
| GRIN2D | NM_000836 | 0.007 | -1.72 | 0.00003 | -2.63 | 0.00005 | -2.68 | 19q13.1-qter |
